# Fifteen-Year Longitudinal Changes in Peripapillary Retinal Nerve Fiber Layer Thickness in Multiple Sclerosis

**DOI:** 10.3390/jcm15156072

**Published:** 2026-08-04

**Authors:** Francisco Gonzalez, Diego de Dios, Jose Manuel Abalo-Lojo

**Affiliations:** 1 Service of Ophthalmology and IDIS, Complejo Hospitalario Universitario de Santiago de Compostela, 15706 Santiago de Compostela, Spain; francisco.gonzalez@usc.es (F.G.); diego.ramon.de.dios.de.santiago@sergas.es (D.d.D.); 2 CIMUS and Department of Surgery and Medico-Surgical Specialties, University of Santiago de Compostela, 15782 Santiago de Compostela, Spain

**Keywords:** optical coherence tomography, multiple sclerosis, peripapillary retinal fiber layer, longitudinal study

## Abstract

**Background/Objectives:** To evaluate changes in peripapillary retinal nerve fiber layer (pRNFL) thickness in patients with multiple sclerosis (MS) and healthy controls over a 15-year period. **Methods:** This is a retrospective, observational, longitudinal, case-control study. The study included 53 patients with relapsing-remitting MS and 44 age-matched healthy controls. pRNFL thickness was measured using optical coherence tomography (OCT) at baseline (2010) and 15 years later (2025) across 12 peripapillary sectors. Eyes were categorized into three groups: healthy control eyes (Control), eyes from MS patients without a history of optic neuritis (MSnoON), and eyes from MS patients with a history of optic neuritis (MS+ON). Median and interquartile range (IQR) were used for descriptive statistics. Non-parametric tests were applied for statistical analysis. **Results:** At baseline, the median pRNFL thickness was 94.8 µm in Control eyes, 86.3 µm in MSnoON eyes, and 69.5 µm in MS+ON eyes. After 15 years, the values were 88.5 µm, 80.8 µm, and 59.5 µm, respectively. Intragroup comparisons between 2010 and 2025 showed statistically significant reductions in pRNFL thickness across all three groups (Wilcoxon test, *p* < 0.05). Intergroup comparisons revealed significant differences among the three groups at both time points (Mann–Whitney test, *p* < 0.05). In all cases, Control eyes exhibited the greatest pRNFL thickness, followed by MSnoON eyes, and then MS+ON eyes. pRNFL thinning was not uniform across all peripapillary sectors. In MSnoON eyes, the temporo-superior and infero-nasal sectors appeared more resistant to fiber loss, whereas in MS+ON eyes, all sectors demonstrated significant thinning. **Conclusions:** Significant pRNFL thinning occurs over time in both MS and healthy eyes. Eyes from MS patients, regardless of a history of optic neuritis, exhibited greater loss than healthy controls. Sectoral analysis suggests differential vulnerability among peripapillary regions. The relative differences between Control, MSnoON, and MS+ON eyes were maintained over a 15-year period.

## 1. Introduction

Multiple sclerosis (MS) is a chronic, inflammatory, and autoimmune neurological disorder of the central nervous system, characterized by demyelination, axonal injury, and atrophy, which lead to progressive neurological deficits [1]. MS is typically classified into two major subtypes: relapsing-remitting and primary progressive [2]. Relapsing-remitting MS is the more prevalent phenotype, affecting approximately 85–90% of patients [3], where neurological impairment primarily results from incomplete recovery following relapses [4].

Given that MS can cause substantial long-term disability, there is a critical need for clinically relevant, non-invasive, and objective biomarkers to facilitate early diagnosis and monitor disease progression. The retina and optic nerve are considered extensions of the brain and, therefore, may serve as valuable indicators of neuroaxonal degeneration associated with MS [5]. Their accessibility enables direct visualization and measurement, offering a unique opportunity to study neurodegenerative changes in vivo. The optic nerve, being a white matter tract [6], is particularly susceptible to structural alterations during MS exacerbations.

Optical coherence tomography (OCT) enables high-resolution, cross-sectional imaging of retinal layers, including the peripapillary retinal nerve fiber layer (pRNFL). Numerous studies have demonstrated that MS is associated with pRNFL thinning and that OCT is particularly sensitive in detecting changes related to optic neuritis (ON) [5]. Early OCT investigations established that ON episodes result in significant RNFL alterations [7,8,9]. Moreover, several studies have reported RNFL thinning even in MS patients without a history of ON, compared to healthy controls [9,10,11], suggesting that RNFL thickness may serve as a promising biomarker for early MS detection.

Most previous OCT studies on RNFL changes in MS have been cross-sectional in design [12]. Longitudinal studies with extended follow-up durations are relatively rare and typically span only 4 to 5 years [13,14,15,16]. These are considerably shorter than the follow-up period reported in the present study. Here, we assess changes in pRNFL thickness over a 15-year period in the eyes of healthy controls, as well as in the eyes of patients with relapsing-remitting MS, with and without a history of ON.

## 2. Methods

### 2.1. Patients and Controls

This retrospective, longitudinal, case-control study included 53 patients with relapsing-remitting multiple sclerosis (MS) (18 males, 35 females; mean age in 2010: 39.2 years, range: 18–64), referred to the Ophthalmology Service of the Complejo Hospitalario Universitario de Santiago de Compostela. The control group comprised 44 age-matched healthy subjects with no history of ocular or neurological disease (12 males, 32 females; mean age in 2010: 41.9 years, range: 20–59) (Table 1).

Only participants with OCT examinations available at both baseline (2010) and the 15-year follow-up (2025) were included in the final analysis.

The study was approved by the Ethics Committee of Santiago-Lugo (Reg. No. 2025/222), adhered to the tenets of the Declaration of Helsinki, and was conducted in accordance with local legislation and institutional requirements.

MS diagnosis was established according to the Poser criteria. Episodes of ON were identified through medical record review. These diagnoses had been established during routine clinical care by the treating neurologists and ophthalmologists according to the clinical standards applicable at the time. Patients with comorbid ocular conditions unrelated to ON were excluded. None of the MS patients had other medical conditions relevant to this study. Eyes were considered as having a history of ON if the episode occurred at any time prior to study inclusion.

### 2.2. Retinal Imaging

Spectral-domain OCT peripapillary images were acquired using the Cirrus HD-OCT system (Model 4000, Carl Zeiss Meditec, Inc., Dublin, CA, USA). To minimize technical bias in the assessment of pRNFL thickness, all scans and measurements were performed using the same device and software in both 2010 and 2025.

**Figure 1:**
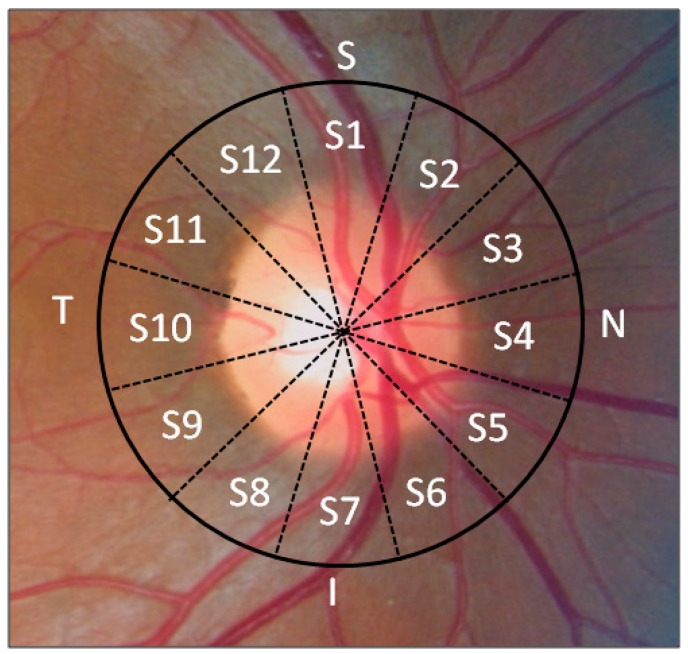
pRNFL sectors analyzed in this study. The image shows the optic nerve head of the right eye and illustrates the division of the surrounding retina into 12 peripapillary sectors (S1 to S12). To ensure anatomical correspondence between left and right eyes, the sectors of the left eye were mirrored horizontally along a vertical axis, such that sector S4 corresponded to the nasal side and sector S10 to the temporal side in both eyes. S = superior, N = nasal, I = inferior, T = temporal.

pRNFL thickness was measured using the Optic Disc Cube 200 × 200 protocol (Carl Zeiss Meditec, Inc., Dublin, CA, USA), which captures 200 horizontal B-scans composed of 200 A-scans each, generating a 6 mm square grid centered on the optic disc. Only scans with a signal strength of 7 or higher (maximum possible: 10) and uniform brightness across the scanned area were considered of acceptable quality. The OCT system provides average RNFL thickness values (in µm) for each of 12 peripapillary sectors. For the purposes of this study, the 12 o’clock sector was defined as superior, 3 o’clock as nasal, 6 o’clock as inferior, and 9 o’clock as temporal (Figure 1). To ensure anatomical correspondence between sectors of the left and right eyes for consistent data analysis, left eye sector maps were mirrored across the vertical axis.

### 2.3. Statistical Analysis

Two pRNFL thickness measurements were obtained for all eyes from both control subjects and MS patients, first in 2010 and again in 2025. The thickness of each peripapillary sector was recorded and used for statistical analysis. A total of 193 eyes were included in the study and categorized into three groups (Table 1): control eyes (Control; 88 eyes, 44 subjects); eyes from MS patients without a history of ON (MSnoON; 64 eyes, 39 patients); and eyes from MS patients with a history of ON (MS+ON; 41 eyes, 27 patients).

Statistical analyses were performed using commercially available software (Excel, Microsoft Corp., Redmond, WA, USA; IBM SPSS Statistics V26, Armonk, NY, USA). The Shapiro–Wilk test, selected for its reliability with small sample sizes, was used to assess the normality of each dataset. As most datasets were not normally distributed, descriptive statistics are presented as median and interquartile range (IQR), and nonparametric tests were employed for statistical comparisons.

Intragroup comparisons between measurements from 2010 and 2025 were conducted using the Wilcoxon matched-pairs signed-rank test (two-sided). Intergroup comparisons for both time points were performed using the Mann–Whitney U test (two-tailed). A *p*-value of <0.05 was considered statistically significant for all analyses. Effect sizes (r) were calculated for intra- and inter-group comparisons for the overall changes in pRNFL. No formal adjustment for multiple comparisons was performed because all sector-wise analyses were pre-specified according to the study protocol.

Both eyes were analyzed as independent observational units according to their clinical classification (Control, MSnoON, or MS+ON). No statistical adjustment for within-subject inter-eye correlation was performed.

## 3. Results

Descriptive data of Control, MSnoON, and MS+ON groups are presented in Table 1. Sixty-four eyes from 39 MS patients showed no evidence of ON either prior to or during the study period. Forty-one eyes from 27 relapsing-remitting MS patients had a documented history of ON prior to the study, and 14 of these eyes (from 9 patients) experienced an additional episode of ON during the study period. Therefore, no eye classified as MSnoON at baseline developed a first episode of optic neuritis during follow-up.

### 3.1. Baseline and 15-Year Follow-Up Measurements

Baseline measurements were obtained in 2010. In the Control group, the median pRNFL thickness across all 12 sectors (S1–S12) was 94.8 µm (IQR: 12.6). In the MSnoON group, the median thickness was 86.3 µm (IQR: 15.6), and in the MS+ON group, it was 69.5 µm (IQR: 24.5). Sector-specific values are presented in Table 2.

Follow-up measurements, obtained 15 years later in 2025, showed a median pRNFL thickness across all 12 sectors (S1–S12) of 88.5 µm (IQR: 13.1) in the Control group. The MSnoON and MS+ON groups had median values of 80.8 µm (IQR: 16.8) and 59.5 µm (IQR: 12.0), respectively. Sector-specific values for the 2025 measurements are also presented in Table 2.

### 3.2. Intragroup Comparison: Baseline vs. 15-Year Follow-Up

Intragroup comparisons between baseline and 15-year follow-up measurements were performed using the Wilcoxon signed-rank test. Corresponding *p*-values are reported in Table 3. In the Control group, a statistically significant reduction in pRNFL thickness was observed over 15 years, with an overall decrease of 6.6% (*p* = 0.000, effect size r = 0.59). All sectors showed significant thinning, with reductions ranging from 1.7% to 7.7%. In the MSnoON group, the overall reduction in pRNFL thickness was 6.4% (*p* = 0.000, effect size r = 0.72), with all sectors demonstrating statistically significant thinning (range: 0.8% to 9.8%). In the MS+ON group, a more pronounced overall reduction of 14.4% was observed (*p* = 0.000, effect size r = 0.75). All sectors except S4 (nasal) showed statistically significant thinning, with reductions ranging from 5.8% to 19.2%.

**Figure 2:**
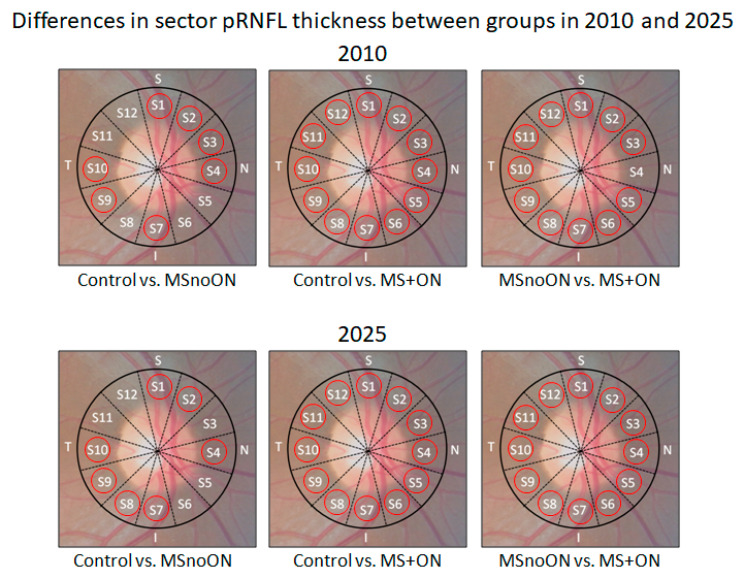
Intergroup comparison of pRNFL thickness by sector for the 2010 and 2025 measurements. Sectors outlined with circles indicate statistically significant differences between groups in the same year (see Table 2, Table 3 and Table 5 for values). S = superior, N = nasal, I = inferior, T = temporal.

Figure 2 illustrates the median pRNFL thickness across all sectors for three groups (Control, MSnoON, MS+ON) at both timepoints (2010 and 2025).

**Figure 3:**
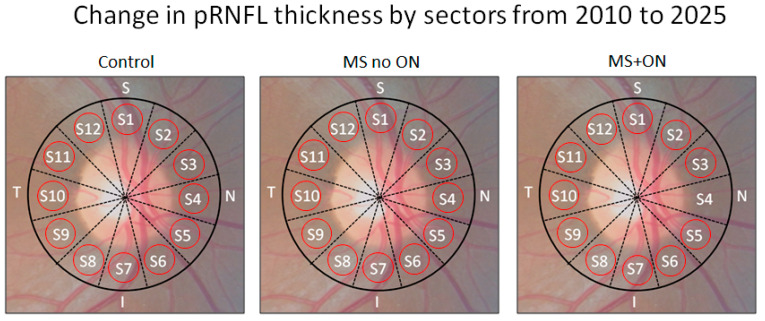
Intragroup changes in pRNFL thickness by sector from baseline (2010) to follow-up (2025). Sectors outlined with circles indicate statistically significant differences between the two time points (See Table 2 for values). S = superior, N = nasal, I = inferior, T = temporal.

Figure 3 shows the intragroup changes in pRNFL thickness over time. The annual rate of thickness reduction was 0.42 µm/year in the Control group, 0.37 µm/year in the MSnoON group, and 0.67 µm/year in the MS+ON group.

**Figure 4:**
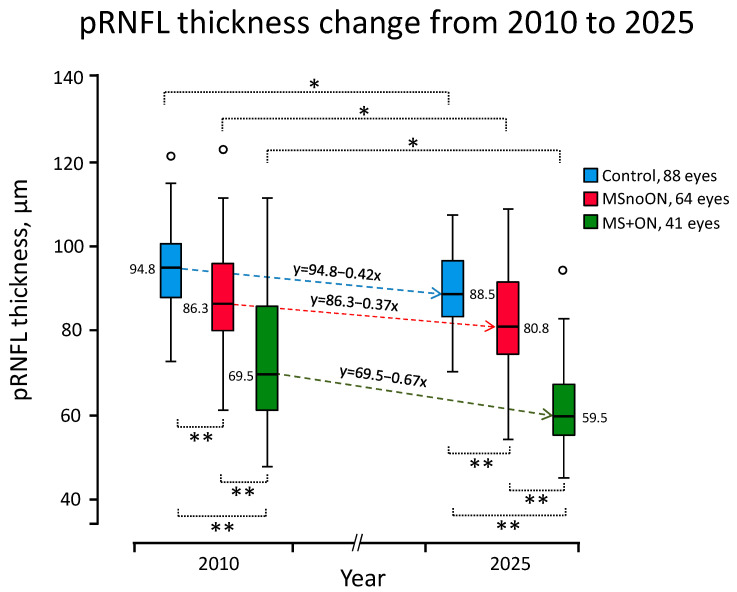
Box plots showing changes in pRNFL thickness in Control, MSnoON, and MS+ON groups from baseline (2010) to follow-up (2025). Intragroup differences between 2010 and 2025 were statistically significant in all groups (* Wilcoxon test, *p* < 0.05). Intergroup differences within the same year were also statistically significant (** Mann–Whitney test, *p* < 0.05). Dotted arrows represent trend lines, with corresponding equations shown (y = pRNFL thickness; x = years). Medians are shown alongside the boxes.

Figure 4 provides a visual summary of data from Table 2, highlighting changes in median pRNFL thickness from 2010 to 2025.

**Figure 5:**
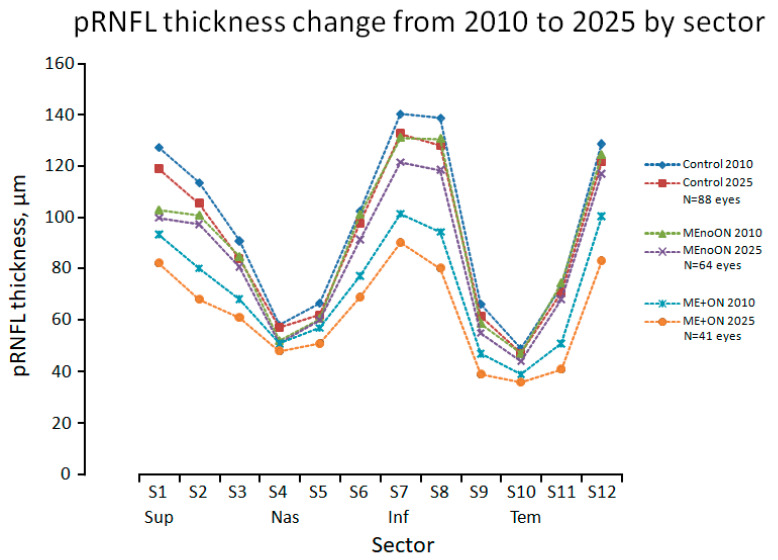
Changes in pRNFL thickness across sectors S1 to S12 from 2010 to 2025 in Control, MSnoON, and MS+ON groups. Each dot represents the median pRNFL thickness for each sector, calculated from all eyes within the respective group. S = superior, N = nasal, I = inferior, T = temporal.

Similarly, Figure 5 displays pRNFL changes for each group and individual sector. Across all groups, nasal and temporal sectors consistently exhibited lower pRNFL thickness compared to superior and inferior sectors. Nearly all sectors showed progressive thinning in pRNFL over time.

### 3.3. Intergroup Comparisons at Baseline (2010)

Intergroup comparisons of baseline pRNFL measurements revealed statistically significant differences among all groups (Mann–Whitney test, *p* < 0.05), as shown in Table 4 and Figure 2.

Comparing Control and MSnoON groups, a 9% reduction in pRNFL thickness was observed in the MSnoON group (*p* < 0.000, effect size r = 0.28). Sector-level analysis showed thicker pRNFL in the Control group across all sectors, though statistical significance was reached in only 7 of the 12 sectors (range: 0% to 18.8%).

Comparisons between Control and MS+ON groups showed markedly thinner pRNFL in the MS+ON group, with an overall reduction of 26.7% (*p* < 0.000, effect size r = 0.58). Sector-specific analysis revealed statistically significant thinning in all 12 sectors (range: 11.9% to 31.7%).

Comparisons between MSnoON and MS+ON groups showed an overall reduction of 19.5% in the MS+ON group (*p* < 0.000, effect size r = 0.46). Sector-specific analysis demonstrated significant thinning in all sectors except S4 (nasal), with reductions ranging from 5.7% to 30.7%.

### 3.4. Intergroup Comparisons at 15-Year Follow-Up (2025)

Intergroup comparisons of the 2025 measurements also revealed statistically significant differences among all groups (Mann–Whitney test, *p* < 0.05), as shown in Table 5 and Figure 2.

**Table 5:** Intergroup differences among Control, MSnoON, and MS+ON groups for year 2025. Median and IQR values are provided in Table 2. In all cases, there was a reduction in pRNFL thickness. N: number of eyes. * Indicates a statistically significant difference between groups (Mann–Whitney test, *p* < 0.05).

2025						
	Control vs. MSnoON		Control vs. MS+ON		MSnoON vs. MS+ON	
Sector	Reduction µm (%)	Mann–Whitney (*p*)	Reduction µm (%)	Mann–Whitney (*p*)	Reduction µm (%)	Mann–Whitney (*p*)
S1–S12	7.7 (8.7)	<0.000 *	29.0 (32.8)	<0.000 *	21.3 (26.4)	<0.000
S1 (Sup.)	19.0 (15.9)	0.000 *	36.5 (30.5)	0.000 *	17.5 (17.4)	0.000 *
S2	8.0 (7.5)	0.002 *	37.0 (34.9)	0.000 *	29.0 (29.6)	0.000 *
S3	3.0 (3.6)	0.074	22.5 (26.6)	0.000 *	19.5 (23.9)	0.000 *
S4 (Nasal)	6.0 (10.3)	0.009 *	9.0 (15.5)	0.000 *	3.0 (5.8)	0.012 *
S5	2.0 (3.2)	0.235	11.0 (17.5)	0.000 *	9.0 (14.8)	0.000 *
S6	6.5 (6.6)	0.054	28.5 (28.9)	0.000 *	22.0 (23.9)	0.000 *
S7 (Inf.)	11.0 (8.3)	0.002 *	42.0 (31.6)	0.000 *	31.0 (25.4)	0.000 *
S8	9.5 (7.4)	0.011 *	47.5 (37.0)	0.000 *	38.0 (31.9)	0.000 *
S9	6.5 (10.4)	0.000 *	22.5 (36.0)	0.000 *	16.0 (28.6)	0.000 *
S10 (Temp.)	3.0 (6.3)	0.001 *	11.0 (22.9)	0.000 *	8.0 (17.8)	0.001 *
S11	2.5 (3.5)	0.130	29.5 (41.3)	0.000 *	27.0 (39.1)	0.000 *
S12	4.5 (3.7)	0.152	38.0 (31.1)	0.000 *	33.5 (28.5)	0.000 *

In comparisons between Control and MSnoON groups, the MSnoON group showed an 8.7% reduction in overall pRNFL thickness (*p* < 0.000, effect size r = 0.33). In the sector-specific analysis, significant thinning was observed in 7 of 12 sectors (range: 6.3% to 15.9%).

The MS+ON group exhibited an overall 32.8% reduction in pRNFL thickness compared to the Control group (*p* < 0.000, effect size r = 0.76). Sector-wise analysis showed statistically significant thinning in all sectors, with reductions ranging from 15.5% to 41.3%.

Comparisons between the MSnoON and MS+ON groups revealed an overall 26.4% reduction in pRNFL in the MS+ON group (*p* < 0.000, effect size r = 0.69). All sectors showed statistically significant thinning (range: 5.8% to 39.1%).

## 4. Discussion

Several longitudinal studies have investigated the time course of pRNFL thickness in MS patients. However, only a few have follow-up periods longer than 4 years [9,13,14,15,16]. Recently, Yam et al. [17] examined pRNFL thickness in patients with relapsing-remitting MS 15 years after the first demyelinating episode but did not compare baseline and follow-up measurements.

To our knowledge, the present study is the first to compare OCT measurements of pRNFL thickness taken at baseline and again 15 years later in both control subjects and MS patients. Importantly, to minimize bias, our scans were obtained using the same OCT device and software. The use of the same OCT device and software throughout the study strengthens the consistency of the longitudinal measurements.

Our results demonstrate a significant loss of retinal ganglion cell axons over time in all three groups. This axonal loss is consistent with the immune-mediated pathophysiology of MS, where inflammatory demyelination leads to neurodegeneration [18,19]. Since pRNFL thickness correlates with the integrity of central nervous system white matter, its measurement by OCT has been recognized as a valuable, non-invasive biomarker for neurodegeneration in MS [20]. Group comparisons in our study demonstrated medium to large effect sizes, indicating substantial separation between the distributions of the compared groups. These findings suggest that the observed between-group differences were not only statistically significant but also of meaningful magnitude, supporting their potential clinical relevance.

### 4.1. Intergroup Comparisons of pRNFL Thickness

We found significant reductions in pRNFL thickness in MS eyes compared to controls, and in MS+ON eyes compared to MSnoON eyes, both at baseline and at the 15-year follow-up (Table 4 and Table 5). Median pRNFL thickness at baseline (2010) was 94.8 µm in controls, 86.3 µm in MSnoON eyes, and 69.5 µm in MS+ON eyes. At follow-up (2025), these values were 88.5 µm, 80.8 µm, and 59.5 µm, respectively. All differences were statistically significant (*p* < 0.001).

Thinning of pRNFL in MS eyes compared with controls is a consistent finding in the literature. For example, Kayhan et al. [21] in a study including 141 eyes of MS patients and 80 control eyes, reported mean pRNFL thickness of 100.6 µm in controls, 86.4 µm in MSnoON eyes, and 77.1 µm in MS+ON eyes. Similarly, Stolowy et al. [22] found mean pRNFL thicknesses of 97.5 µm in controls, 96.3 µm in MSnoON eyes, and 82.6 µm in MS+ON eyes. Najafi et al. [23] reported pRNFL median values of 117.0 µm in control eyes, 106.0 µm in MSnoON eyes, and 85.0 µm in MS+ON eyes. Yam et al. [17] reported that eyes with prior ON had thinner pRNFL (74.3 µm) than unaffected MS eyes (89.2 µm) at 15 years after the first demyelinating event. Abalo-Lojo et al. [9] also found significant differences over a 5-year period, with baseline median values of 96.8 µm (controls), 90 µm (MSnoON), and 74.2 µm, which declined to 94.5, 84.7, and 68.7 µm, respectively. Several additional studies have reported significant reductions of pRNFL thickness in eyes of MS patients compared to healthy controls, but did not differentiate between MSnoON and MS+ON eyes [20,24,25].

Several meta-analyses further confirm these patterns. Filippatou et al. [26] analyzed 170 studies (8542 control eyes, 14,822 MSnoON eyes, 5529 MS+ON eyes) and reported pRNFL thickness reductions of 6.9 µm (control vs. MSnoON), 18.0 µm (control vs. MS+ON), and 10.8 µm (MSnoON vs. MS+ON). El Ayoubi et al. [5] conducted a meta-analysis of 100 studies (2291 to 4494 eyes) and found similar differences: 6.97 µm, 16.44 µm, and 9.74 µm, respectively. In comparison, our study reports greater reductions: 8.5 µm (control vs. MSnoON), 25.3 µm (control vs. MS+ON), and 16.8 µm (MS+ON vs. MSnoON) at baseline, and 7.7 µm, 29.0 µm, and 21.3 µm, respectively, after 15 years.

While absolute values of pRNFL thickness vary across studies, the hierarchical pattern of thickness (Control > MSnoON > MS +ON) is consistent and, in our case, persists over a 15-year period.

### 4.2. Intergroup Comparisons by Sector

Changes in pRNFL thickness were not uniform across peripapillary sectors (Figure 2 and Figure 3, Table 3, Table 4 and Table 5). At baseline, comparisons between Control and MSnoON eyes revealed relative preservation in the temporo-superior (S11–S12) and infero-nasal (S5–S6, S8) sectors, whereas the remaining sectors showed a significant thinning. A similar sectorial pattern persisted at the 15-year follow-up.

In contrast, comparisons between Control and MS+ON eyes showed significant thinning across all sectors at both time-points. MS+ON eyes also exhibited significantly thinner pRNFL compared to MSnoON in all sectors at baseline, except the nasal sector. By 2025, however, a significant thinning was observed in all sectors. Najafi et al. [23] conducted pairwise comparisons between healthy eyes and MS eyes with and without ON. They reported decreased total, superior, and inferior pRNFL thickness in both MS groups. However, because their analysis was by quadrants rather than by individual sectors, direct comparisons with our sector-based results are limited.

### 4.3. pRNFL Changes over Time

This study provides data on pRNFL changes in healthy eyes and in MS eyes, with and without a history of ON, over a 15-year period. Pairwise comparisons between baseline and endpoint measurements showed significant thinning in all three groups (see Table 3). This fiber loss was observed across nearly all sectors, except for the nasal sector (S4) in MS+ON eyes. The total reduction in pRNFL thickness over this 15-year follow-up was 6.3 µm (6.6%) in Control eyes, 5.5 µm (6.4%) in MSnoON eyes, and 10.0 µm (14.4%) in MS+ON eyes.

It should also be noted that 14 eyes experienced recurrent optic neuritis during the 15-year follow-up period. Because of the limited number of recurrent cases and the retrospective design of the study, a separate subgroup analysis was not feasible. Nevertheless, recurrent optic neuritis may have contributed to the greater pRNFL thinning observed in the MS+ON group. Future prospective studies including larger cohorts of patients with recurrent optic neuritis are needed to better characterize the cumulative effect of repeated inflammatory episodes on long-term retinal neuroaxonal degeneration.

The annualized rate of fiber loss in this period was higher in MS+ON eyes (0.67 µm/year) compared to healthy (0.42 µm/year) and MSnoON eyes (0.37 µm/year). The slightly lower rate in MSnoON eyes compared to controls is unexpected and may reflect a selective vulnerability, where more susceptible fibers are lost early in the disease, leaving behind a population of more resilient axons. However, this interpretation remains speculative and should be confirmed in future longitudinal studies with more frequent OCT assessments. The annualized rates of fiber loss reported in this study were calculated from OCT measurements obtained at only two time points (baseline and the 15-year follow-up). Therefore, these values should be interpreted as average rates over the entire follow-up period rather than evidence of a linear constant progression. Because no intermediate OCT examinations were available, the temporal trajectory of pRNFL thinning could not be determined. It is possible that retinal thinning occurred at variable rates during follow-up, with periods of accelerated loss after inflammatory activity or optic neuritis followed by relative stabilization. Future longitudinal studies incorporating serial OCT examinations at multiple intermediate time points are needed to better characterize the dynamics of retinal neurodegeneration in multiple sclerosis.

Our findings are broadly consistent with those of previous longitudinal studies with shorter follow-up periods. For example, Gernet et al. [27] conducted a three-year study and reported a significantly greater annual pRNFL loss in MS patients (0.73%) compared to controls (0.13%). Similarly, Toscano et al. [28] evaluated 419 patients with relapsing-remitting MS and observed a decline in pRNFL thickness from 94.6 µm to 91.9 µm in the right eye and from 94.3 µm to 91.6 µm in the left eye over three years, corresponding to a thinning rate of 0.90 µm/year in both eyes. These rates are notably higher than those observed in our study (0.37 µm/year in MSnoON eyes and 0.67 µm/year in MS+ON eyes). Furthermore, Toscano et al. [28] reported that a pRNFL thickness ≤ 88.0 µm was an independent predictor of cognitive impairment and disability progression in MS patients. Other studies have also reported higher thinning rates in MSnoON eyes. For instance, De Angelis et al. [29] followed 212 MS patients over a two-year period and found annual thinning rates of 0.69 µm/year in MS+ON eyes and 0.97 µm/year in MSnoON eyes. Differences in reported thinning rates across studies may be attributed to variations in follow-up duration and the timing of measurements. It is likely that pRNFL loss is not constant over time and may be more pronounced in the early stages of the disease. Supporting this, Küchlin et al. [30] reported that in eyes with isolated ON, global pRNFL thickness declined by 2.1 µm/week during the first four weeks, by 1.7 µm/week through week 16, and then showed 0.2 µm/week by week 26.

Notably, progressive pRNFL thinning is observed in MS eyes even in the absence of a history of ON. This thinning correlates with greater brain atrophy, increased visual and global disability, and reduced quality of life [31]. Several authors have highlighted the utility of OCT in detecting neurodegeneration along the visual pathway, suggesting that OCT can be considered a reliable and reproducible method to evaluate disability progression [27] and gray matter damage [20] in MS patients.

Although the present study focuses on multiple sclerosis, retinal thinning has also been described in several other neurodegenerative disorders, including Alzheimer’s disease, Parkinson’s disease, and amyotrophic lateral sclerosis. In these conditions, OCT-derived retinal measurements have been proposed as non-invasive biomarkers reflecting neuroaxonal degeneration and disease burden. The growing body of evidence across different neurological disorders supports the concept that retinal imaging may provide valuable insights into central nervous system pathology. Our findings further reinforce the potential role of pRNFL assessment as part of this expanding field of retinal biomarkers in neurology [32,33].

Although our study focused exclusively on patients with relapsing-remitting multiple sclerosis, retinal structural abnormalities are not specific to this disease and may also occur in other neurological disorders affecting the visual pathway, including hereditary optic neuropathies such as Leber hereditary optic neuropathy (LHON). In selected clinical scenarios, LHON may coexist with multiple sclerosis, a condition known as Harding’s disease, further emphasizing the importance of considering alternative or overlapping diagnoses when interpreting retinal imaging findings. Therefore, OCT findings should always be interpreted within the appropriate clinical context and in conjunction with a comprehensive neurological and ophthalmological evaluation, particularly in complex diagnostic scenarios [34].

The rate of retinal nerve fiber layer (RNFL) thickness reduction from 2010 to 2015 in our control group, across all peripapillary sectors (S1–S12), was 0.42 µm/year. This value is similar to that reported by Leung et al. [35], who reported a reduction rate of 0.52 µm/year in healthy subjects over a follow-up period of 30 months, with thinning rates of 1.35 µm/year and 1.25 µm/year in the superior and inferior quadrants, respectively, with no detectable differences in the nasal and temporal quadrants. These regional differences across the quadrants are consistent with our findings. We found the highest reduction rates in the superior (mean across S1–S3, 0.51 µm/year) and inferior (mean across S7–S9, 0.49 µm/year) quadrants, whereas the lowest reduction rates were observed in the nasal (mean across S4–S6, 0.22 µm/year) and temporal (mean across S10–S12, 0.28 µm/year) quadrants. The significant RNFL thinning observed in the nasal and temporal quadrants in our study may be explained by the longer duration of follow-up and the lower age of our control subjects (41.9 ± 10.3 years) compared with those in the study by Leung et al. (56 ± 6.69 years).

### 4.4. Study Strengths and Limitations

The main strength of our study is the long interval between baseline and endpoint assessments of pRNFL thickness, which spanned 15 years. To our knowledge, this study represents the longest OCT-based longitudinal evaluation of pRNFL thickness in patients with multiple sclerosis reported to date. A second strength lies in the consistency of the imaging methodology: to minimize device-dependent bias, all OCT measurements were obtained using the same device and software. However, several limitations should be acknowledged.

The retrospective design led to the inclusion of patients based on data availability, resulting in a non-randomized sample. Previous episodes of optic neuritis (ON) were identified through medical record review. Although ON diagnoses had been established during routine clinical care by experienced neurologists and ophthalmologists, the absence of standardized prospective confirmation using predefined clinical, electrophysiological, and neuroimaging criteria may have introduced a degree of classification bias. Furthermore, important clinical and functional variables, including visual evoked potentials (VEPs), Expanded Disability Status Scale (EDSS) scores, disease duration, relapse burden, and magnetic resonance imaging (MRI) findings, were not available in a standardized manner for all participants or at comparable time points throughout the follow-up period. Consequently, reliable longitudinal correlation analyses between pRNFL thinning and functional visual pathway impairment, disability progression, or inflammatory disease activity could not be performed.

In this study, both eyes were analyzed as independent units. Although this approach may overestimate the effective sample size because of the intra-subject correlation between fellow eyes, it was adopted given the exploratory nature of the study and because the analyses focused on eye-specific characteristics. This should be acknowledged as a methodological limitation when interpreting the findings. In addition, no formal correction for multiple comparisons was applied. Therefore, the large number of sector-wise statistical comparisons may have increased the risk of type I error, and these findings should be interpreted with appropriate caution.

Although healthy controls were age-matched at baseline and underwent the same follow-up period, age was not included as a covariate in the statistical analyses. Future prospective longitudinal studies should incorporate age-adjusted statistical models to better distinguish physiological retinal aging from disease-related neuroaxonal loss.

OCT image analysis is susceptible to artifacts and variability in image processing and segmentation, which may arise from inaccuracies in software algorithms [36] or changes in ocular media transparency. The study included only patients with relapsing-remitting MS and did not compare findings across different MS subtypes. We did not account for potential confounding factors such as tobacco or alcohol use, subclinical comorbidities, or treatment history, including disease-modifying therapies (DMTs), treatment duration, treatment changes during follow-up, or exposure to medications with potential optic neurotoxicity, all of which may have influenced pRNFL thinning rate. Our study population may not fully represent the broader MS population, as individuals with more advanced disease may have been underrepresented.

Despite these limitations, our findings are consistent with those of previously published studies. The results reported here, together with evidence from other studies, are particularly relevant given the need to identify reliable biomarkers in MS is a critical step toward improving clinical decision-making and prognosis. Fleisher et al. [37] recently examined the prognostic value of both single and multimodal biomarker combinations for predicting disease progression in MS patients and found that OCT-derived measurements were among those with the highest discriminative accuracy.

### 4.5. Future Directions

Future prospective longitudinal studies integrating OCT with standardized clinical assessments, electrophysiological testing, neuroimaging, and treatment-related variables are warranted to further establish the role of retinal imaging as a biomarker of disease progression in multiple sclerosis. A multimodal approach combining structural, functional, and clinical biomarkers may improve disease monitoring, prognostic assessment, and therapeutic decision-making.

## 5. Conclusions

This study provides the longest OCT-based longitudinal evaluation of peripapillary retinal nerve fiber layer (pRNFL) thickness in patients with relapsing-remitting multiple sclerosis reported to date. Significant pRNFL thinning was observed over the 15-year follow-up period in both healthy controls and patients with multiple sclerosis, with substantially greater thinning in eyes with a history of optic neuritis. Sectoral analysis demonstrated that retinal neuroaxonal loss is not uniformly distributed across the peripapillary retina. These findings support the potential role of OCT as a non-invasive tool for the long-term assessment of neuroaxonal degeneration and reinforce the value of pRNFL thickness as a potential biomarker of disease progression in multiple sclerosis.

## Figures and Tables

**Table 1:** Descriptive data for the control, MsnoON, and MS+ON groups. Age is presented as mean, standard deviation, and range.

Group	Subjects/ Patients	Eyes	M/F	Age in 2010, Years Mean (SD) Range
Control	44	88	12/32	41.9 (10.3) 20–59
MSnoON	39	64	14/25	39.2 (8.7) 22–59
MS+ON	27	41	10/17	39.0 (8.6) 18–59

**Table 2:** Changes in pRNFL thickness (µm) from baseline (2010) to the year 2025 in eyes from the Control, MSnoON, and MS+ON groups. S1 to S12 indicate peripapillary sectors. Medians and IQR for S1–S12 were calculated using the median values of all sectors of each individual eye. Median and IQR for individual sectors (S1, S2, …) were calculated from each sector of each eye. N: number of eyes. P: number of patients.

	Control (N = 88, P = 44)				MSnoON (N = 64, P = 39)				MS+ON (N = 41, P = 27)			
	2010		2025		2010		2025		2010		2025	
Sector	Median	IQR	Median	IQR	Median	IQR	Median	IQR	Median	IQQ	Median	IQR
S1–S12	94.8	12.6	88.5	13.1	86.3	15.6	80.8	16.8	69.5	24.5	59.5	12.0
S1 (Sup.)	127.5	46.3	119.5	25.6	103.5	44.3	100.5	32.5	94.0	34.0	83.0	32.0
S2	114.0	24.3	106.0	19.6	101.5	30.5	98.0	24.3	81.0	36.0	69.0	23.0
S3	91.5	23.3	84.5	15.4	85.5	21.5	81.5	23.3	69.0	32.0	62.0	25.0
S4 (Nasal)	59.0	15.0	58.0	8.8	53.0	14.5	52.0	16.0	52.0	12.0	49.0	10.0
S5	67.5	22.3	63.0	12.9	61.5	18.0	61.0	19.0	58.0	12.0	52.0	10.0
S6	103.0	33.3	98.5	19.4	102.0	33.8	92.0	28.3	78.0	34.0	70.0	25.0
S7 (Inferior)	140.5	35.0	133.0	24.9	131.5	35.3	122.0	31.3	102.0	44.0	91.0	30.0
S8	139.0	28.0	128.5	19.9	131.0	29.8	119.0	24.5	95.0	39.0	81.0	30.0
S9	67.0	17.3	62.5	14.7	59.5	20.5	56.0	18.3	48.0	19.0	40.0	13.0
S10 (Temp.)	50.0	10.0	48.0	9.0	48.0	9.3	45.0	12.3	40.0	17.0	37.0	15.0
S11	75.0	12.0	71.5	12.4	75.0	21.0	69.0	22.5	52.0	27.0	42.0	19.0
S12	129.0	29.0	122.0	18.7	125.0	28.0	117.5	24.8	101.0	31.0	84.0	30.0

**Table 3:** Intragroup statistical differences in pRNFL between baseline (2010) and year 2025. Median and IQR values are provided in Table 2. * Indicates statistically significant difference (Wilcoxon test, *p* < 0.05).

2010 vs. 2025						
	Control (N = 88)		MSnoON (N = 64)		MS+ON (N = 41)	
	Reduction µm (%)	Wilcoxon (*p*)	Reduction µm (%)	Wilcoxon (*p*)	Reduction µm (%)	Wilcoxon (*p*)
S1–S12	6.3 (6.6)	0.000 *	5.5 (6.4)	0.000 *	10.0 (14.4)	0.000 *
S1 (Sup.)	8.0 (6.3)	0.000 *	3.0 (2.9)	0.000 *	12.0 (11.7)	0.000 *
S2	8.0 (7.0)	0.000 *	3.5 (3.4)	0.000 *	7.0 (14.8)	0.000 *
S3	7.0 (7.7)	0.000 *	4.0 (4.7)	0.000 *	7.0 (10.1)	0.000 *
S4 (Nasal)	1.0 (1.7)	0.000 *	1.0 (1.9)	0.022 *	3.0 (5.8)	0.066
S5	4.5 (6.7)	0.015 *	0.5 (0.8)	0.021 *	6.0 (10.3)	0.002 *
S6	4.5 (4.4)	0.011 *	10.0 (9.8)	0.000 *	8.0 (10.3)	0.011 *
S7 (Inferior)	7.5 (5.3)	0.004 *	9.5 (7.2)	0.000 *	11.0 (10.8)	0.000 *
S8	10.5 (7.6)	0.000 *	12.0 (9.2)	0.000 *	14.0 (14.7)	0.000 *
S9	4.5 (6.7)	0.000 *	3.5 (5.9)	0.000 *	8.0 (16.7)	0.003 *
S10 (Temp.)	2.0 (4.0)	0.000 *	3.0 (6.3)	0.000 *	3.0 (7.5)	0.004 *
S11	3.5 (4.7)	0.000 *	6.0 (8.0)	0.000 *	10.0 (19.2)	0.000 *
S12	7.0 (5.4)	0.000 *	7.5 (6.0)	0.000 *	17.0 (16.8)	0.000 *

**Table 4:** Intergroup differences between Control, MSnoON, and MS+ON groups for year 2010. Median and IQR values are provided in Table 2. In all cases, there was a reduction in pRNFL thickness. N: number of eyes. * Indicates a statistically significant difference between groups (Mann–Whitney test, *p* < 0.05).

2010						
	Control vs. MSnoON		Control vs. MS+ON		MSnoON vs. MS+ON	
Sector	Reduction µm (%)	Mann–Whitney (*p*)	Reduction µm (%)	Mann–Whitney (*p*)	Reduction µm (%)	Mann–Whitney (*p*)
S1–S12	8.5 (9.0)	<0.000 *	25.3 (26.7)	<0.000 *	16.8 (19.5)	<0.000 *
S1 (Sup.)	24.0 (18.8)	0.000 *	33.5 (26.3)	0.000 *	9.5 (9.2)	0.011 *
S2	12.5 (11.0)	0.004 *	33.0 (28.9)	0.000 *	20.5 (20.2)	0.000 *
S3	6.0 (6.6)	0.100 *	22.5 (24.6)	0.000 *	16.5 (19.3)	0.000 *
S4 (Nasal)	6.0 (10.2)	0.001 *	7.0 (11.9)	0.000 *	1.0 (1.9)	0.167
S5	6.0 (8.9)	0.133	9.5 (14.1)	0.000 *	3.5 (5.7)	0.007 *
S6	1.0 (1.0)	0.529	25.0 (24.3)	0.000 *	24.0 (23.5)	0.000 *
S7 (Inf.)	9.0 (6.4)	0.047 *	38.5 (27.4)	0.000 *	29.5 (22.4)	0.000 *
S8	8.0 (5.8)	0.063	44.0 (31.7)	0.000 *	36.0 (27.5)	0.000 *
S9	7.5 (11.2)	0.001 *	19.0 (28.4)	0.000 *	11.5 (19.3)	0.002 *
S10 (Temp.)	2.0 (4.0)	0.029 *	10.0 (20.0)	0.000 *	8.0 (16.7)	0.009 *
S11	0.0 (0.0)	0.652	23.0 (30.7)	0.000 *	23.0 (30.7)	0.000 *
S12	4.0 (3.1)	0.252	28.0 (21.7)	0.000 *	24.0 (19.2)	0.000 *

## Data Availability

Data from human subjects were used in this study.

## Abbreviations

**IQR** = interquartile range; **MS** = multiple sclerosis; **MS+ON** = multiple sclerosis eyes with optic neuritis; **MSnoON** = multiple sclerosis eyes with no optic neuritis; **OCT** = optical coherence tomography; **ON** = optic neuritis; **pRNFL** = peripapillary retinal nerve fiber layer; **RNFL** = retinal nerve fiber layer; **S1**–**S12** = papillary sectors 1 to 12.
